# Botulism Sequelae: A Systematic Review

**DOI:** 10.1093/ofid/ofaf773

**Published:** 2025-12-12

**Authors:** Mark Kosenko, Veronika Rogozhina, Tamerlan Erdniev, Armen K Shakaryan, Dmitry Tumurov, Ekaterina Ligskaya, Svetlana Gadetskaya, Alina Eremeeva, Margarita Andreeva, Maria Pyatnitskaya, Ekaterina Pazukhina, Alan Asmanov, Danilo Buonsenso, Luis Felipe Reyes, Timothy R Nicholson, Alla Guekht, Daniel Munblit

**Affiliations:** Department of Paediatrics and Paediatric Infectious Diseases, Institute of Child's Health, I.M. Sechenov First Moscow State Medical University, Sechenov University, Moscow, Russia; Department of Paediatrics and Paediatric Infectious Diseases, Institute of Child's Health, I.M. Sechenov First Moscow State Medical University, Sechenov University, Moscow, Russia; Department of Paediatrics and Paediatric Infectious Diseases, Institute of Child's Health, I.M. Sechenov First Moscow State Medical University, Sechenov University, Moscow, Russia; Federal State Autonomous Scientific Institution “Chumakov Federal Center for Research and Development of Immune-and-Biological Products of the Russian Academy of Sciences” (Institute of Poliomyelitis) (FSASI “Chumakov FSC R&D IBP RAS”), Moscow, Russia; Department of Children's Infectious Diseases, Faculty of Pediatrics, Pirogov Russian National Research Medical University, Moscow, Russia; Moscow Research and Clinical Center for Neuropsychiatry, Moscow, Russia; Department of Paediatrics and Paediatric Infectious Diseases, Institute of Child's Health, I.M. Sechenov First Moscow State Medical University, Sechenov University, Moscow, Russia; Department of Paediatrics and Paediatric Infectious Diseases, Institute of Child's Health, I.M. Sechenov First Moscow State Medical University, Sechenov University, Moscow, Russia; Department of Paediatrics and Paediatric Infectious Diseases, Institute of Child's Health, I.M. Sechenov First Moscow State Medical University, Sechenov University, Moscow, Russia; University of British Columbia, Vancouver, Canada; Erasmus School of Health Policy & Management, Rotterdam, Netherlands; Centre for Cancer Screening, Prevention and Early Detection, Wolfson Institute of Population Health, Queen Mary University of London, London, UK; Veltischev Scientific Research Clinical Institute of Pediatrics and Children Surgery, Pirogov Russian National Research Medical University, Moscow, Russia; Department of Woman and Child Health and Public Health, Fondazione Policlinico Universitario A. Gemelli IRCCS, Rome, Italy; Unisabana Center for Translational Science, School of Medicine, Universidad de La Sabana, Chia, Colombia; Institute of Psychiatry, Psychology & Neuroscience, King's College London, London, UK; Moscow Research and Clinical Center for Neuropsychiatry, Moscow, Russia; Department of Neurology, Neurosurgery and Medical Genetics, Pirogov Russian National Research University, Moscow, Russia; Department of Paediatrics and Paediatric Infectious Diseases, Institute of Child's Health, I.M. Sechenov First Moscow State Medical University, Sechenov University, Moscow, Russia; Care for Long Term Conditions Division, Florence Nightingale Faculty of Nursing, Midwifery and Palliative Care, King's College London, London, UK

**Keywords:** botulism, post-infectious sequelae, quality of life, systematic review, *Clostridium botulinum*

## Abstract

**Background:**

Botulism is a life-threatening neuroparalytic disease caused by botulinum neurotoxins. While its acute phase has been extensively studied, long-term sequelae following recovery remain insufficiently explored. This systematic review aims to comprehensively assess and synthesize available evidence on post-botulism sequelae to improve understanding and guide future research.

**Methods:**

A systematic search was conducted in MEDLINE, EMBASE via Ovid, and Web of Science from inception to 24 June 2024. Eligible studies included observational studies, case series, and case reports describing post-recovery symptoms in individuals diagnosed with foodborne, wound, or infant botulism, excluding iatrogenic cases. The risk of bias was assessed using the Critical Appraisal Skills Programme (CASP) checklists.

**Results:**

Out of 340 screened records, 9 studies met inclusion criteria, comprising 2 case-control studies (*n* = 230 botulism cases, *n* = 669 controls) and 7 cohort studies (*n* = 185 botulism cases). Most studies reported some short- and/or long-term consequences of botulism. Among 3 studies homogeneously reporting sequelae symptomatology, the most frequently reported long-term symptoms included fatigue (66.2%, range 47.9%–84.6%), limitations in vigorous activities (55.8%, range 47.6%–64.0%), general weakness (57.1%, range 43.1%–76.9%), and dyspnea (42.9%, range 18.0%–92.3%). In some patients, psychosocial dysfunction persisted longer than physical impairments, over the 6-year period post intoxication. Two studies provided comparative data with control groups, demonstrating significantly higher prevalence of fatigue, weakness, and impaired health perception in botulism survivors. Additionally, 14 case reports and case series (*n* = 43 individuals) reported similar patterns with dyspnea, fatigue, and autonomic dysfunction among the most reported sequelae.

**Conclusions:**

This systematic review highlights significant long-term sequelae among botulism survivors, particularly fatigue, respiratory impairment, and psychosocial dysfunction. While recovery trajectories suggest improvement over time, persistent symptoms may impact quality of life. Standardized outcome measures and longitudinal studies are needed to elucidate the burden of post-botulism sequelae further and inform clinical management strategies.

## Teaser

First systematic review of observational studies and case reports finds that some botulism survivors face prolonged fatigue, respiratory limitations and psychosocial impairment, with normal spirometry but reduced exercise capacity, emphasising the importance of recognising and managing post-botulism sequelae in care.

## Graphical Abstract

**Graphical Abstract:**
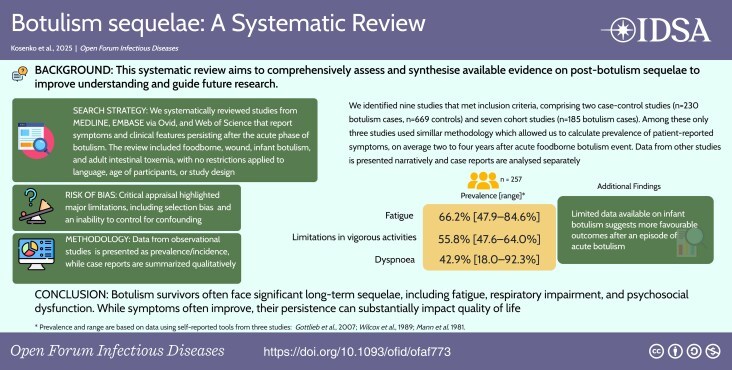
This graphical abstract is also available at Tidbit: https://tidbitapp.io/tidbits/botulism-sequelae-a-systematic-review?utm_campaign=tidbitlinkshare&utm_source=ITP

Botulism is a life-threatening disease caused by toxins produced by botulinum neurotoxins-producing clostridia [1]. Based on the route, this condition may vary in its type, causing foodborne, wound, infant, iatrogenic botulism, and adult intestinal toxemia [1]. The botulism is a relatively rare condition with a limited number of outbreaks worldwide. The incidence varies across different geographical regions. In 2019, the Center for Disease Control and Prevention in the United States reported 215 cases of botulism [2] while 82 confirmed cases of botulism were found across EU/EEA countries in 2020 [3]. One of the largest outbreaks of botulism in decades occurred in July 2024 in Moscow, Russia, with over 400 individuals affected by the disease [4]. Botulism is a rare but serious illness that usually requires hospital admission and immediate treatment and is associated with prolonged hospital stay with the most severe patients requiring intubation and mechanical ventilation [1].

Short- and long-term sequelae after the acute phase of infectious disease were reported across different conditions, such as Middle East respiratory syndrome (MERS), Ebola, dengue, chikungunya virus infection, and others [5]. Although post-infectious sequelae are not new; they received the most attention following the COVID-19 pandemic, with a major interest in the long COVID-19/post COVID-19 condition [6], which has been named as a potential global burden [7, 8]. Post-infectious sequelae are associated with quality of life impairment and require medical attention [8, 9], which may be hampered by the lack of resources and attention to the problem.

The acute phase of botulism has already been extensively studied, with several systematic reviews collating evidence [10–12], but only a limited number of studies have evaluated botulism sequelae [13–17] with no systematic evaluation conducted to date. This systematic review aimed to comprehensively assess existing evidence on the sequelae of botulism to improve understanding of the topic and provide the basis for further research.

## METHODS

This systematic review follows the recommendations set forth by the Preferred Reporting Items for Systematic Reviews and Meta-Analyses (PRISMA) statement [18]. The review was registered with the National Institute for Health Research's PROSPERO 2024 CRD42024568513 Available from: https://www.crd.york.ac.uk/prospero/display_record.php?ID=CRD42024568513.

### Search Strategy and Selection Criteria

MEDLINE, EMBASE via Ovid, and Web of Science were searched for relevant peer-reviewed studies from the inception to 24 June 2024, when the search was conducted. Additionally, reference lists of eligible articles, relevant review articles, and gray literature sources were searched to identify additional relevant studies not identified in the original database search. Search strategy combined controlled vocabulary and free-text terms related to long-term outcomes and sequelae (eg, “long-term outcome*,” “persisting impairment*,” “permanent sequelae*,” “lasting symptom*”), outcome assessment (including patient-reported outcomes), and botulism (including foodborne, wound, and infant botulism). The complete search syntax for each database is provided in the Supplementary Data.

Two authors (V. R. and T. E.) independently screened titles and abstracts and reviewed full texts of potentially eligible records using Covidence systematic review software (Veritas Health Innovation). Any disagreements between the screeners were resolved via consensus or additional reviewer involvement (M. K. and D. M.).

*Eligibility criteria*: All studies reporting symptoms and clinical features following recovery from the acute phase of botulism. Due to the known paucity of available data, case reports and case series were also included but analyzed and presented independently from the original study results.

*Population*: Humans (irrespective of age) with diagnosed botulism.

*Exposure*: Four natural exposure routes of botulism as defined by the CDC: foodborne, wound, infant, and adult intestinal toxemia. Iatrogenic botulism is excluded due to its fundamentally different exposure mechanism.

*Outcome*: Prevalence/incidence and clinical characteristics of the sequelae.

### Data Extraction

A data extraction form was developed in Microsoft Excel and pre-piloted using 5 randomly selected studies. The form was revised based on the pilot results to ensure clarity and comprehensiveness. Two reviewers (V. R., T. E.) independently extracted information from all potentially relevant articles, with discrepancies resolved via discussions with the third reviewer (M. K.). Studies in languages other than English were translated using a combination of automated translation tools and reviewed by fluent speakers to ensure accuracy and fidelity to the original text.

The data extracted from eligible papers included study characteristics, type of botulism, participant characteristics, information about follow-up, sequelae type, measurement instrument/clinical definitions used, and incidence/prevalence.

### Risk of Bias Assessment

Based on the study's design, the quality of included studies was assessed using Critical Appraisal Skills Programme (CASP) Checklists for cohort studies [19] or case-control studies [20]. These tools consist of 11 and 12 questions respectively, divided into 3 sections: validity of results, results appraisal, and applicability of results [19, 20]. Two researchers (V. R., T. E.) independently conducted the risk of bias assessment, and all discrepancies were resolved by a third reviewer (M. K.).

### Data Analysis

Given the heterogeneity in study design, reported sequelae, and measurement instruments, a meta-analysis was not feasible. Instead, a narrative synthesis approach was employed in accordance with the Synthesis Without Meta-analysis (SWiM) guideline [19]. The data were analyzed descriptively, with key findings summarized in tabular form, stratifying studies based on study design, population, and outcome measures. The data on symptoms reported homogeneously across the studies were plotted for illustrative purposes.

For observational studies, we reported the prevalence and incidence of botulism sequelae using absolute frequencies and percentages, along with ranges where possible. For studies that allowed direct comparisons between botulism survivors and controls, we extracted and reported odds ratios (ORs) with 95% confidence intervals (CIs), if available. Functional and psychosocial impairment measures were presented using standardized scoring scales as reported in the original studies. Since there are no standardized criteria for post-infectious sequelae, we considered any new or long-lasting symptom or clinical feature after recovery from the acute phase of botulism as a post-botulism sequelae irrespective of time frame.

For case reports and case series, due to the descriptive nature of these studies, results were summarized qualitatively and presented separately from observational data. A structured data extraction table was developed to categorize reported sequelae by type, duration of follow-up, and study setting.

All statistical analyses were performed using R software (Version 4.4.1, R Foundation for Statistical Computing, Vienna, Austria). When available, reported statistical significance levels (*P*-values) were extracted and included in the synthesis to provide additional context for interpretation.

## RESULTS

**Figure 1.**
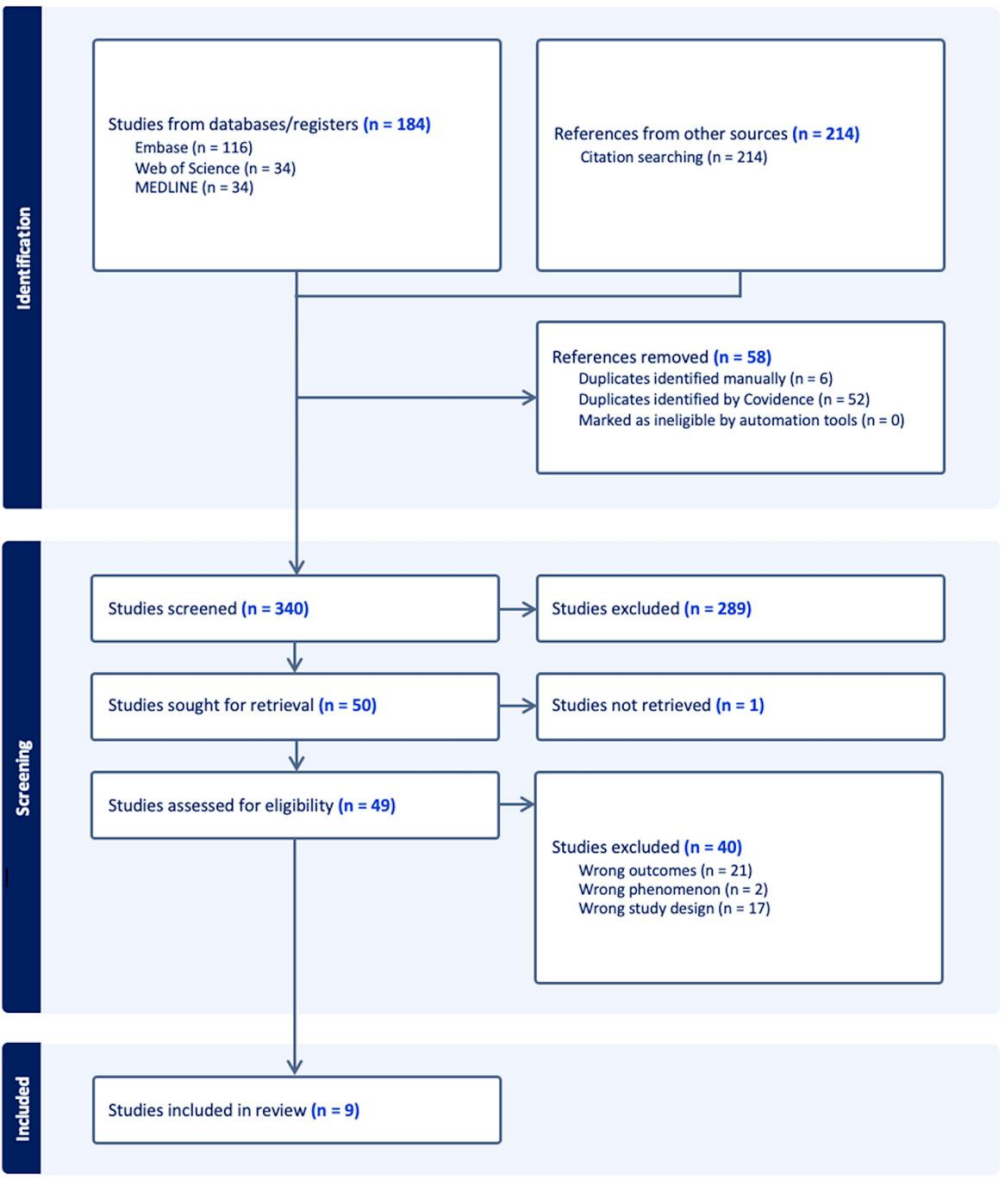
Preferred Reporting Items for Systematic Reviews and Meta-Analyses (PRISMA) flow chart of the systematic search and included studies.

The systematic literature search resulted in a total of 340 records. The full text for one of the studies was not identified, and the authors did not respond to our inquiries [20]. Nine manuscripts met pre-defined inclusion criteria for this systematic review (Figure 1). Two case-control studies [14, 15] assessed 230 cases of foodborne botulism (FBB) and 669 control subjects, while 7 cohort studies [13, 16, 17, 21–24] covered 185 cases of botulism, with 5 of the latter being prospective. The main characteristics of the included studies are presented in Table 1. Seven studies [13–17, 22, 24] included individuals presenting with FBB, and 2 [21, 23] focused on infant botulism (IB). The diagnosis of botulism was primarily based on epidemiological and laboratory findings. However, a substantial proportion of cases were diagnosed on clinical grounds alone. This reliance on clinical diagnosis introduces a limitation to our analysis. The potential for misclassification is underscored by the work of Gottlieb et al [15], whose study, including the largest participant cohort, explicitly noted the lack of a standardized case definition for botulism in Georgia during the study period. Sample sizes of included studies ranged from 8 to 217. Out of 9 studies included, only 5 reported botulism sequelae investigation as a primary aim [13–17]. Two studies [13, 17] included overlapping patient datasets following a botulism outbreak in New Mexico (April 1978), during which 34 persons developed clinical botulism.

**Table 1.** Main Characteristics of Included Studies

Author, Year	Study Design	Country	Population	Time to Follow-Up	Outcome Assessment Method	Outcomes
**Foodborne botulism**						
**Gottlieb et al, 2007** [**15]**	Case-control study	Georgia	217 hospitalized patients (10–72 y.o.) at least 6 months after botulism and matched 656 controls 34 cases were laboratory confirmed (33 involved toxin type B, and 1 involved toxin type E)	4.3 years (range 0.6–6.4 years)	Interview (using modified 36-item short form questionnaire)	- 68% of survivors reported worse health post-botulism compared to 17% of controls (OR: 17.6, 95% CI: 10.9–28.4) - 49% of survivors rated their health as “fair” or “poor” versus 25% of controls (OR: 5.0, 95% CI: 3.2–7.6) - Persistent symptoms: fatigue (48%), weakness (43%), dizziness (31%), dry mouth (32%) - Functional limitations: 64% reported difficulty with vigorous activities versus 38% of controls (OR: 5.0, 95% CI: 3.2–7.7) - Psychosocial impact: survivors were significantly less likely to feel energetic (OR: 0.3, 95% CI: 0.2–0.5) and were more likely to report nervousness (OR: 2.8, 95% CI: 1.8–4.4) and sleep disturbances (OR: 1.8, 95% CI: 1.1–3.1) - Predictors of worse long-term health: mechanical ventilation, older age, and region of residence
**Wilcox et al, 1989** [**14]**	Case-control study	Canada	13 cases of type B botulism outbreak (mean age ± SD = 35 ± 13) and 13 age- and sex-matched control subjects	2 years	Questionnaire, specialized tests for respiratory function and exercise tolerance	- Persistent symptoms at 2 years: dyspnea on exertion (all ventilated patients), fatigue (85%), and weakness (77%) - Respiratory function: lung function tests had normalized, but 4/13 patients (31%) showed inspiratory muscle weakness (PImax < 65% predicted) - Exercise limitation: maximal oxygen consumption (VO₂max) and maximal workload were significantly reduced compared to controls (*P* < 0.05) - Breathing pattern: botulism patients had a more rapid, shallow breathing pattern during exercise and higher dyspnea scores at given ventilation levels (*P* < 0.05) - Autonomic dysfunction: persistent dry mouth, constipation, impotence, and resting tachycardia, suggesting prolonged cholinergic blockade - Exercise limitation multifactorial: some patients had residual respiratory muscle weakness, but most were limited by reduced cardiovascular fitness, leg fatigue, or motivation rather than ventilatory constraints
**Cohen et al, 1988** [**16]**	Prospective cohort study	United States	28 adults admitted to a hospital and ICU (12 required mechanical ventilation) after type A botulism outbreak	4, 8, 12, 18, 24, and 36 months	Interview during home visits (using the Sickness Impact Profile—SIP)	- Persistent psychosocial dysfunction: at 3 years post-botulism, psychosocial dysfunction remained significantly elevated compared to pre-illness levels, whereas physical dysfunction had largely resolved - SIP scores: during hospitalization, physical impairment was twice as severe as psychosocial impairment, but by 4 months, the scores were nearly identical. Psychosocial impairment remained elevated beyond 3 years - Comparison of ventilated versus non-ventilated patients: mechanical ventilation was associated with higher physical dysfunction scores during hospitalization and at 4 months, but no significant differences in psychosocial dysfunction at any time point. This suggests that psychosocial sequelae were not solely dependent on physical disability - Emotional impact: patients experienced early hospitalization anxiety, followed by long-term depression, anger, and heightened vulnerability. Support from spouses, religion, and peer groups played a major role in coping - Clinical implications: psychosocial dysfunction was long-lasting and independent of initial disease severity, highlighting the need for mental health support and anticipatory guidance in recovery
**Schmidt-Nowara et al, 1983** [**13]**	Prospective cohort study	United States	34 patients (11 required mechanical ventilation) after type A botulism outbreak; 22 participated in follow-up	1 year	Questionnaire, specialized tests for respiratory function	- Recovery slowed after 6 months, despite initial improvement - At 1 year, all but 2 patients had residual symptoms, with fatigue (68%) as the most disabling, often triggered by minimal exertion - Ventilated patients had significantly more symptoms, including fatigue and dyspnea (50%). 29% experienced severe dyspnea, requiring them to stop for breath while walking - Pulmonary function was normal in most patients, except for reduced maximal midexpiratory flow (FEF 25–75) in ventilated patients
**Mann et al, 1981** [**17]**	Prospective cohort study	United States	27 hospitalized adults after type A botulism outbreak	9, 13, 24 months	Questionnaire and interview	- Individual symptoms persisted for at least 9 months in some patients - While only 13% of patients still complained of difficulty swallowing 9 months after onset, 62% continued to report shortness of breath - Ocular (double vision, blurred vision) and bulbar symptoms (difficulty speaking clearly, difficulty swallowing) tended to resolve more rapidly than autonomic (constipation, dry mouth, dry eyes) or muscular (general weakness, shoulder/arm weakness, shortness of breath, exercise intolerance) symptoms
**Townes et al, 1996** [**22]^a^**	Retrospective cohort study	United States	8 adult patients (20–48 y.o.) after type A botulism outbreak in mixed setting	6 months	Neurologic examination	At follow-up visits 6 months later, the results of neurologic examinations of 4 patients were normal
**Boccagni et al, 2021** [**24]^a^**	Prospective cohort study	Italy	18 male adults (47 ± 8.4 years) after botulism outbreak	73–80 days	Neurologic examination, neurophysiological studies using electromyography	- Clinically, all follow-up participants had diplopia, while half showed weakness in the upper and lower limbs and reduced deep tendon reflexes - Compound muscle action potential (CMAP) amplitudes decreased from 100% in the acute phase to 20% in the early post-acute phase and 17% in the late post-acute phase - Abnormal postexercise CMAP facilitation dropped from 100% (acute) to 40% (early post-acute) and 0% (late post-acute) - Pathological responses to high-frequency repetitive nerve stimulation (HFRNS) declined from 80% (acute) to 50% (early post-acute) and 8% (late post-acute). Small MUAPs decreased from 100% (acute/early post-acute) to 50% (late post-acute)
**Infant botulism**						
**Vanella De Cuetos et al, 2011** [**23]^a^**	Retrospective cohort study	Argentina	49 cases of type A infant botulism demanding admission in ICU and mechanical ventilation	3 months	NR	Severe residual hypotonia and laryngeal stenosis were noticed in 4 and 1 untreated infants, respectively, and none of the EqBA-treated patients presented these sequelae. Hypotonic patients recovered by 90 days after discharge from the hospital
**Tseng-Ong et al, 2007 [21]^a^**	Retrospective cohort study	United States	44 hospitalized infants (20–247 days old), 39 with verified types A and B infant botulism	NR	Physical examination	All infants survived, and all who had follow-up examinations after discharge had normal strength, motor development, and neurologic status when they were last seen

^a^Study was not included in the analysis and its outcomes are discussed narratively [13–17, 23–25].

### Foodborne Botulism

Two [22, 24] out of 7 studies on FBB reported limited data on botulism sequelae as this has not been the primary study outcome. Townes et al [22]. investigated 8 patients with botulism and followed 4 of them for 6 months after convalescence. All 4 underwent neurologic examination with no signs or symptoms reported [22]. Boccagni et al [24] followed 4 patients out of 18 male adults following FBB outbreak for a short period of time of 2 and a half months. Half of the individuals showed weakness in the upper and lower limbs and reduced deep tendon reflexes; diplopia, dysphagia, hypophonia, dysphonia, and dysarthria were noticed in one patient each [21, 23, 24].

Five remaining studies assessed FBB sequelae as a primary outcome. One of them, by Cohen et al [16], used the Sickness Impact Profile (SIP) [25, 26], a health status measure designed to assess the impact of sickness and disability on an individual's daily functioning to longitudinally assess sequelae in 28 adults, focusing on physical, psychosocial, and independent categories dysfunction over the 3-year period post botulism. The overall SIP mean decreased steadily over 3 years, starting from 50.44 during hospitalization to 3.95 at 36 months. Physical impairment was severe during hospitalization (mean = 63.68) but declined sharply, reaching 1.09 by 36 months. Conversely, psychosocial impairment was initially lower (mean = 33.21) but decreased more gradually, remaining at 6.53 at 36 months. At 4 months post-admission, physical and psychosocial scores intersected, with physical impairment continuing its rapid decline while psychosocial dysfunction persisted longer.

**Figure 2.**
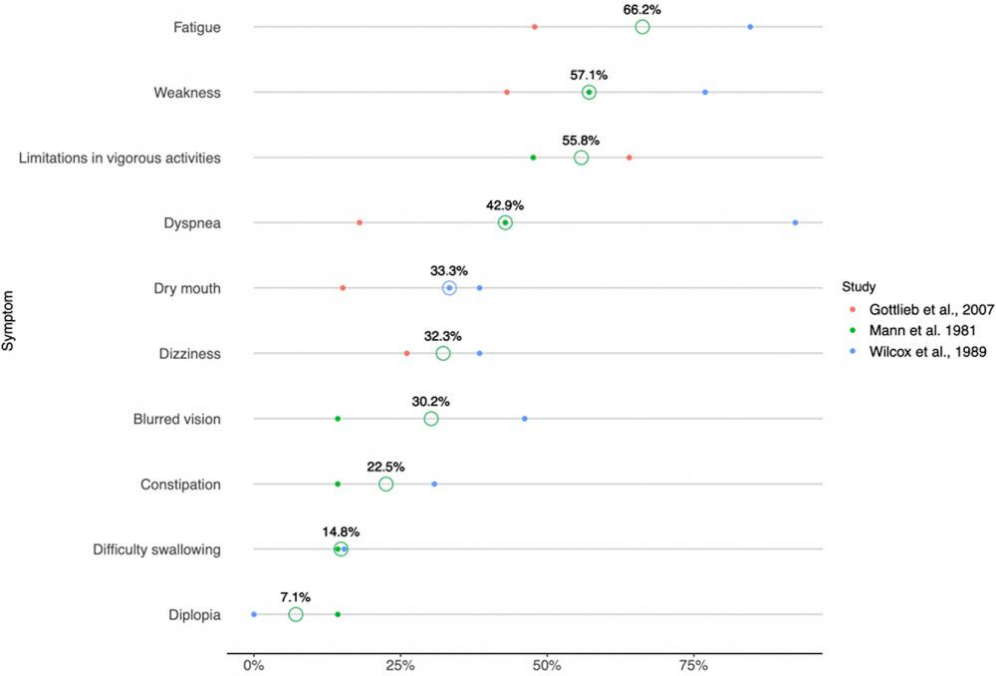
Prevalence of patient-reported symptoms, on average 2–4 years after acute foodborne botulism event. Data from Schmidt-Nowara et al^13^ study is not shown as authors followed the same cohort of patients as Mann et al^17^.

Out of 7 studies on FBB 4 [13–15, 17] used comparable methodology and approach to symptomatology reporting and categorization (Supplementary Data), which allowed to plot these data (Figure 2). The most frequently reported symptoms on average 2–4 years after FBB were fatigue (median 66.2%, range 47.9–84.6), limitations in vigorous activities (55.8%, 47.6–64.0), general weakness (57.1%, 43.1–76.9), and dyspnea (42.9%, 18.0–92.3) [13–15, 17].

Only 2 studies compared sequelae between FBB survivors and control subjects. Gottlieb et al [15] found that patients were more likely than controls to report worse health, whether they had required mechanical ventilation (OR = 33.4, 95% CI: 11.3–98.9) or not (OR 16.3, 9.6–28.1). Forty-nine percent of patients rated their current health as “fair” or “poor,” compared to 25% of controls (OR = 5.0, 3.2–7.6). Experience of botulism was associated with more frequent fatigue (OR 3.6, 2.4–5.5) and weakness (OR 3.2, 2.1–4.8), with limitations in vigorous activities (eg, running and climbing stairs) (OR 32.3, 1.6–3.5). Patients also reported feeling less energetic (OR 0.3, 0.2–0.5) or happy (OR 0.3, 0.2–0.5) and were more likely to feel nervous (OR 2.8, 1.8–4.4) and disturbed sleep (OR 1.8, 1.1–3.1). Another study by Wilcox et al [14] focused on respiratory functioning and exercise tolerance 2 years post FBB; despite improvements, all 13 patients reported dyspnea with minimal or moderate exertion, unlike age- and sex-matched controls, where only 2 reported dyspnea with moderate exertion. Pulmonary function tests showed no significant differences between groups, and no restrictive abnormalities were found in participants. Respiratory muscle strength was generally comparable, though 4 patients had a PImax < 65% predicted, indicating inspiratory muscle weakness. Exercise capacity was reduced in botulism survivors, with 6 achieving < 80% of predicted VO₂max, and they showed a rapid, shallow breathing pattern during exercise.

### Infant Botulism

Of the 9 identified studies, only 2 by Vanella De Cuetos et al [23] and Tseng-Ong et al [21] provided information on IB sequelae. Both studies primarily focused on clinical presentation, diagnosis, and treatment rather than long-term outcomes. In a 3-month follow-up of 49 infants, Vanella De Cuetos et al [23] reported severe residual hypotonia in 2 patients and laryngeal stenosis in one. In contrast, Tseng-Ong et al [21] reported no sequelae in their cohort of 44 infants. However, neither study specified the number of infants assessed at follow-up nor the duration of follow-up for Tseng-Ong et al [21]. Consequently, the scarcity and heterogeneity of the data precluded any meaningful analysis or conclusions regarding sequelae following IB.

The CASP critical appraisal was conducted and the major limitations associated with the study design and conduct were the following: selection bias related to investigation of a group of individuals following a single botulism outbreak [13, 14, 17, 24]. Most studies did not adjust for confounding factors, which may be attributable to the small sample sizes and the fact that many studies were conducted few decades ago, when methodological standards for observational research were less rigorous. The full CASP assessment for the included studies is available in the Supplementary Data.

**Figure 3.**
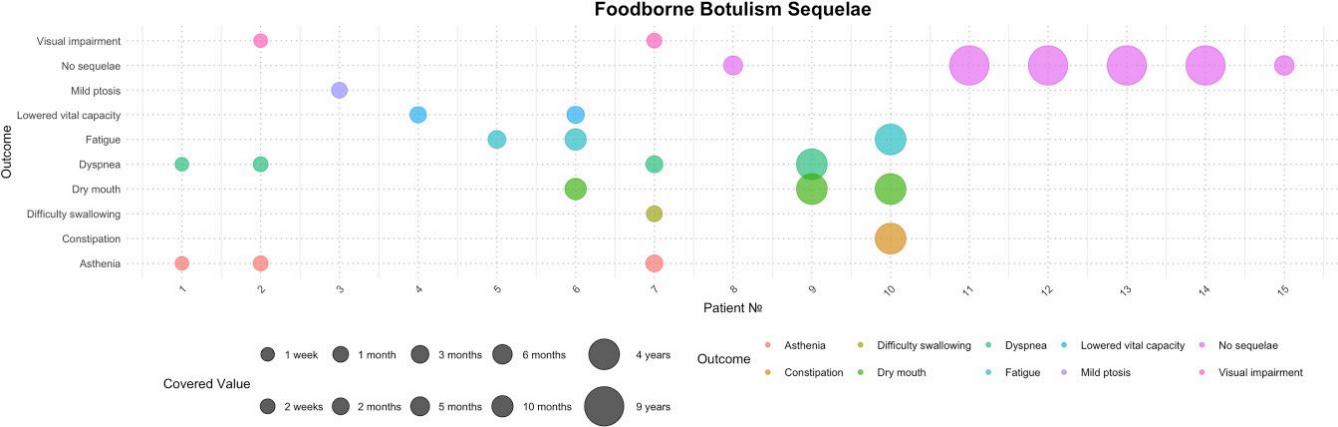
Patient-reported symptoms described in case report and case series studies after acute foodborne botulism event.

We have additionally collated the data from case reports and case series presenting patients with follow-up examinations after botulism. Fourteen manuscripts covered clinical scenarios on 43 individuals (Supplementary Data). These included 17 individuals after FBB [24, 27, 28, 29, 30, 31] infants after IB [32–38], a child after wound botulism [28], and an individual after, supposedly, adult intestinal toxemia as reported by the authors [39]. The most prevalent long-term symptoms (Figure 3) among individuals after FBB event were dyspnea (24%), fatigue (18%), asthenia (18%), and dry mouth (18%), including severe dryness of the mouth which has not resolved even 4 years after FBB [27–31]. Common symptoms reported were consistent with the outcomes of the studies included in the analysis [13–15, 17].

Patients after IB reported in case reports and case series had generally favorable outcomes [32–38] with most infants (83.3%) reporting no sequelae during follow-up visits. The most prevalent symptom following IB was weakness (12.5%) noticed during the first 2 months after convalescence.

## DISCUSSION

This systematic review provides the first comprehensive synthesis of long-term sequelae following botulism, highlighting persistent physical, respiratory, and psychosocial impairments among survivors. Our findings indicate that while many patients recover from the acute phase, a substantial proportion experience prolonged symptoms, including fatigue, dyspnea, muscle weakness, and limitations in daily activities. These sequelae align with post-infectious syndromes described in other bacterial and viral illnesses, suggesting shared mechanisms underlying long-term recovery impairments.

Long-term complications following infectious diseases have been well documented, particularly in the context of post-viral syndromes. The persistence of fatigue, dyspnea, and autonomic nervous system dysfunction in botulism survivors is similar to findings from studies on post-COVID-19 conditions, post-sepsis syndrome, and other infections with peripheral nervous system involvement. We draw cross-disease parallels at the level of shared clinical phenotypes—fatigue, exertional dyspnea, and autonomic and psychosocial dysfunction, while emphasizing that the pathophysiology differs materially. In botulism, persistent symptoms follow a toxin-mediated presynaptic blockade of acetylcholine at the neuromuscular junction with prolonged autonomic cholinergic dysfunction; recovery likely reflects gradual restoration of neuromuscular transmission with possible incomplete regeneration and dysautonomia, rather than ongoing organ inflammation. Consistent with this mechanism, long-term cohorts show normal spirometry yet inspiratory muscle weakness and reduced VO₂max with rapid, shallow breathing on exertion, features pointing to neuromuscular limitation. By contrast, analogous symptom clusters after other infections (eg, severe viral pneumonias, sepsis) often arise from inflammatory organ injury or neuroinflammatory pathways. Our comparisons contextualize what patients experience across conditions while clarifying that botulism's long-term morbidity is rooted in toxin-mediated neuromuscular and autonomic dysfunction, not the inflammatory or structural organ damage more typical elsewhere.

Similar to post-polio syndrome, which manifests years after acute poliovirus infection with progressive muscle weakness, fatigue, and respiratory dysfunction [40], botulism survivors experience residual neuromuscular impairment. This is expected, given that *Clostridium botulinum* toxin exerts its primary effect by blocking acetylcholine release at the neuromuscular junction [41]. Unlike post-polio syndrome, however, botulism sequelae appear to improve over time rather than progressively worsen, although psychosocial dysfunction and reduced exercise tolerance may persist.

Long-term respiratory dysfunction has been observed in various infections requiring mechanical ventilation, although it is usually found in patients facing severe infections impacting respiratory system. For example, severe COVID-19 pneumonia leads to persistent dyspnea, reduced lung function, and exertional intolerance months after hospital discharge [42, 43]. Similarly, survivors of severe acute respiratory syndrome (SARS) and Middle East respiratory syndrome (MERS) reported exertional breathlessness and persistent fatigue for years [44]. These patterns are comparable to botulism survivors, in whom residual dyspnea on exertion, inspiratory muscle weakness, and reduced maximal oxygen consumption (VO₂max) were prominent findings in long-term follow-up studies [14, 15]. However, the origin of these sequelae is likely to be very different and the data on botulism survivors is limited.

Post-infectious fatigue is a recognized syndrome, often reported after viral and bacterial illnesses. Survivors of botulism, as seen in our review, frequently experience persistent fatigue and exercise intolerance. This aligns with findings from post-sepsis syndrome, where up to 50% of patients report long-term fatigue, cognitive dysfunction, and impaired health-related quality of life [45]. Additionally, fatigue and autonomic dysfunction have been widely documented in post-Ebola and post-dengue patients, reinforcing the likelihood of post-infectious neuroinflammatory pathways contributing to prolonged recovery [46, 47]. Beyond fatigue, psychosocial impairment appears to be a major consequence of botulism. Cohen et al identified persisting psychological distress and social limitations in survivors, even after the resolution of physical symptoms [16]. This finding is consistent with psychological sequelae observed in post-COVID-19 and post-sepsis populations, where high rates of depression, anxiety, and PTSD have been reported [47]. The persistence of neuropsychological dysfunction following botulism warrants further investigation, as neurotoxin-mediated effects on the autonomic nervous system may play a role in long-term psychosocial distress.

The mechanisms underlying prolonged symptoms in botulism survivors remain speculative but may involve long-term neuromuscular junction dysfunction, neuroplasticity changes, and persistent autonomic dysregulation. Studies on botulinum neurotoxin recovery suggest that while neuromuscular transmission may return over time, compensatory synaptic changes and incomplete regeneration could contribute to lingering symptoms. Additionally, the role of post-infectious neuroinflammation, as seen in post-viral syndromes, remains an area for future research.

While this systematic review provides a comprehensive synthesis of existing evidence on long-term sequelae following botulism, several limitations must be acknowledged. A major limitation of this review is the heterogeneity across included studies, particularly in terms of study design, population characteristics, follow-up duration, and outcome assessment methods. The included studies varied significantly in sample size, ranging from small case series to larger cohort and case-control studies, making direct comparisons challenging. Additionally, the lack of standardized outcome measures across studies limits the ability to draw firm conclusions regarding the true prevalence and severity of botulism sequelae. Several of the included studies focused on specific botulism outbreaks, which may introduce selection bias. Patients from these outbreaks may not represent all botulism cases globally, particularly given the geographic variation in clinical management, access to botulinum antitoxin, and supportive care. Many studies relied on retrospective data collection and self-reported outcomes, which may introduce recall bias and subjectivity in symptom reporting. Self-reported symptoms, such as fatigue, dyspnea, and psychosocial distress, may be influenced by individual perception and recall, particularly in studies with long follow-up periods. Objective functional assessments (eg, pulmonary function tests, electromyography, or cognitive evaluations) were not consistently performed, limiting the ability to correlate subjective complaints with physiological dysfunction. While 2 studies included control groups for comparison, the majority did not, making it difficult to determine whether all of the reported symptoms were specific to botulism or reflective of general post-illness recovery patterns. The absence of age- and sex-matched controls in most studies limits the ability to attribute observed sequelae solely to botulism, as other comorbid conditions or preexisting health factors may contribute to symptoms.

This systematic review highlighted significant long-term sequelae among botulism survivors, with some individuals experiencing fatigue, respiratory impairment, and psychosocial dysfunction persisting months to years after acute illness. These findings draw parallels with other post-infectious syndromes, reinforcing the need for standardized outcome measures and longitudinal studies to characterize post-botulism morbidity better. Given the significant burden of post-botulism sequelae, clinicians should be aware of potential long-term neuromuscular and psychosocial dysfunction in affected patients. Structured rehabilitation programs, pulmonary rehabilitation, and psychological support may benefit survivors, particularly those experiencing persistent fatigue, exercise intolerance, or psychosocial distress. Future studies should focus on prospective, long-term follow-up of botulism survivors to better understand recovery trajectories and identify optimal interventions.

## Supplementary Material

ofaf773_Supplementary_Data

## Supplementary Data

Supplementary materials are available at *Open Forum Infectious Diseases* online. Consisting of data provided by the authors to benefit the reader, the posted materials are not copyedited and are the sole responsibility of the authors, so questions or comments should be addressed to the corresponding author.
