# Supplementary material for: Botulism Sequelae: A Systematic Review
Source: Open Forum Infect Dis. 2025 Dec 12;13(1):ofaf773. doi: 10.1093/ofid/ofaf773 (PMC12798721; doi:10.1093/ofid/ofaf773)
Supplement: ofaf773_Supplementary_Data [file ofaf773_supplementary_data.docx]

**Supplementary material**

**Botulism infection sequelae: A Systematic Review**

Mark Kosenko ^1^*, Veronika Rogozhina ^1^*, Tamerlan Erdniev ^1^*, Armen K Shakaryan ^2,3^, Dmitry Tumurov ^4^, Ekaterina Ligskaya ^1^, Svetlana Gadetskaya^1^, Alina Eremeeva ^1^, Margarita Andreeva ^5^, Maria Pyatnitskaya ^6^, Ekaterina Pazukhina ^7^, Alan Asmanov ^8^, Danilo Buonsenso ^9^, Luis Felipe Reyes ^10^, Timothy R Nicholson ^11^, Alla Guekht ^4^, Daniel Munblit ^1,12^*

1. Department of Paediatrics and Paediatric Infectious Diseases, Institute of Child’s Health, I.M. Sechenov First Moscow State Medical University, Sechenov University, Moscow, Russia
2. Federal State Autonomous Scientific Institution "Chumakov Federal Center for Research and Development of Immune-and-Biological Products of the Russian Academy of Sciences" (Institute of Poliomyelitis) (FSASI "Chumakov FSC R&D IBP RAS"), Moscow, Russia
3. Department of Children's Infectious Diseases, Faculty of Pediatrics, Pirogov Russian National Research Medical University, Moscow, Russia
4. Moscow Research and Clinical Center for Neuropsychiatry, Moscow, Russia
5. University of British Columbia, Vancouver, Canada
6. University of Rotterdam, Rotterdam, Netherlands
7. Centre for Cancer Screening, Prevention and Early Detection, Wolfson Institute of Population Health, Queen Mary University of London, London, United Kingdom
8. Veltischev Scientific Research Clinical Institute of Pediatrics and Children Surgery, Pirogov Russian National Research Medical University, Moscow, Russia.
9. Department of Woman and Child Health and Public Health, Fondazione Policlinico Universitario A. Gemelli IRCCS, Rome, Italy
10. Unisabana Center for Translational Science, School of Medicine, Universidad de La Sabana, Chia, Colombia
11. Institute of Psychiatry, Psychology & Neuroscience, King's College London, London, United Kingdom
12. Care for Long Term Conditions Division, Florence Nightingale Faculty of Nursing, Midwifery and Palliative Care, King's College London, London, United Kingdom

Table S1. Search strategy.

| EMBASE via Ovid | 1. ("long-term outcome*" or "long-term prognos*" or "long-term sequelae*" or "long-term effect*" or "long-term deficit*" or "long-term problem*" or "long-term symptom*" or "long-term impairment*" or "long-term defect*" or "persisting defect*" or "persisting deficit*" or "persisting problem*" or "persisting impairment*" or "persisting symptom*" or "persisting effect*" or "persisting outcome*" or "persisting sequelae*" or "permanent sequelae*" or "permanent outcome*" or "permanent effect*" or "permanent symptom*" or "permanent impairment*" or "permanent problem*" or "permanent defect*" or "permanent deficit*" or "lasting deficit*" or "lasting defect*" or "lasting problem*" or "lasting impairment*" or "lasting symptom*" or "lasting outcome*" or "lasting sequelae*" or "ongoing sequelae*" or "ongoing prognos*" or "ongoing outcome*" or "ongoing symptom*" or "ongoing impairment*" or "ongoing problem*" or "ongoing defect*" or "ongoing deficit*" or "ongoing effect*" or "lasting effect*" or "outcome score*" or "outcome measure*" or "long-term follow-up" or "ongoing follow up" or "sequelae*").ab,kf,ti.  2. outcome assessment/  3. patient-reported outcome/  4. 1 or 2 or 3  5. botulism.ab,kf,ti.  6. foodborne botulism/ or botulism/ or wound botulism/ or infant botulism/  7. 5 or 6  8. 4 and 7 |
| --- | --- |
| MEDLINE via Ovid | 1. ("long-term outcome*" or "long-term prognos*" or "long-term sequelae*" or "long-term effect*" or "long-term deficit*" or "long-term problem*" or "long-term symptom*" or "long-term impairment*" or "long-term defect*" or "persisting defect*" or "persisting deficit*" or "persisting problem*" or "persisting impairment*" or "persisting symptom*" or "persisting effect*" or "persisting outcome*" or "persisting sequelae*" or "permanent sequelae*" or "permanent outcome*" or "permanent effect*" or "permanent symptom*" or "permanent impairment*" or "permanent problem*" or "permanent defect*" or "permanent deficit*" or "lasting deficit*" or "lasting defect*" or "lasting problem*" or "lasting impairment*" or "lasting symptom*" or "lasting outcome*" or "lasting sequelae*" or "ongoing sequelae*" or "ongoing prognos*" or "ongoing outcome*" or "ongoing symptom*" or "ongoing impairment*" or "ongoing problem*" or "ongoing defect*" or "ongoing deficit*" or "ongoing effect*" or "lasting effect*" or "outcome score*" or "outcome measure*" or "long-term follow-up" or "ongoing follow up" or "sequelae*").ab,kf,ti.  2. Outcome Assessment, Health Care/  3. Patient Reported Outcome Measures/  4. 1 or 2 or 3  5. botulism.ab,kf,ti.  6. Botulism/  7. 5 or 6  8. 4 and 7 |
| Web-of-Science | (ALL=("long-term outcome*" or "long-term prognos*" or "long-term sequelae*" or "long-term effect*" or "long-term deficit*" or "long-term problem*" or "long-term symptom*" or "long-term impairment*" or "long-term defect*" or "persisting defect*" or "persisting deficit*" or "persisting problem*" or "persisting impairment*" or "persisting symptom*" or "persisting effect*" or "persisting outcome*" or "persisting sequelae*" or "permanent sequelae*" or "permanent outcome*" or "permanent effect*" or "permanent symptom*" or "permanent impairment*" or "permanent problem*" or "permanent defect*" or "permanent deficit*" or "lasting deficit*" or "lasting defect*" or "lasting problem*" or "lasting impairment*" or "lasting symptom*" or "lasting outcome*" or "lasting sequelae*" or "ongoing sequelae*" or "ongoing prognos*" or "ongoing outcome*" or "ongoing symptom*" or "ongoing impairment*" or "ongoing problem*" or "ongoing defect*" or "ongoing deficit*" or "ongoing effect*" or "lasting effect*" or "outcome score*" or "outcome measure*" or "long-term follow-up" or "ongoing follow up" or "sequelae*")) AND ALL=(botulism) |

Table S2. Assessment of sequelae in patients following botulism infection reported in case-reports and case-series studies.

| **Patient №** | **Follow up period** | **First author, Year** | **Country** | **Sex & Age** | **Setting** | **Sequelae type** | **Infection control method** |
| --- | --- | --- | --- | --- | --- | --- | --- |
| **Foodborne botulism** | | | | | | | |
| **1** | 7 days* | Jalda et al., 2016 | Spain | F, 60 years | Hospital, ICU | Asthenia and dyspnoea on exertion for up to 7 days post-discharge | Clinical presentation; Epidemiological data;  Stool and serum samples |
| **2** | 7 - 15 days* | Jalda et al., 2016 | Spain | M, 33 years | Hospital, ICU | Blurred vision which persisted for 7 days; asthenia and dyspnoea on exertion both of which persisted for 15 days | Clinical presentation; Epidemiological data |
| **3** | 1 month* | Oliveira et al., 2022 | Brazil | M, 5 years | Hospital | A global improvement of muscle strength, mild ptosis, and ability to walk without support | Clinical presentation; Serum sample; EMG |
| **4** | 46 days* | Paust, 1971 | USA | F, 22 years | ICU | Lowered vital capacity | Serum samples; Epidemiological data; Clinical presentation |
| **5** | 15 - 90 days* | Jalda et al., 2016 | Spain | M, 64 years | Hospital, ICU | Blurred vision which persisted for 15 days; dysphagia persisting for 30 days; asthenia and dyspnoea on exertion for up to 60 days post-discharge. Patient required outpatient physical therapy for three months | Clinical presentation; Epidemiological data |
| **6** | 3 months* | Paust, 1971 | USA | F, 48 years | ICU | Fatigue which resolved during next few months, and essentially normal pulmonary functions | Serum samples; Epidemiological data; Clinical presentation |
| **7** | 5 months** | Cherington, 1974 | USA | F, 20 years | Hospital | No sequelae | Clinical presentation; Serum sample; EMG |
| **8** | 6 months* | Sicurella et al., 2021 | Italy | F, 53 years | Hospital | No sequelae | Stool sample, Electromyography, Repetitive nerve stimulation,  Blood sample |
| **9** | 2,5 - 10 months* | Paust, 1971 | USA | M, 49 years | ICU | Lowered vital capacity 2.5 months after discharge. Sequelae 10 months after the onset of the illness are a persistent dryness of the mouth and fatigue, but the patient was able to work full time by then | Serum samples; Epidemiological data; Clinical presentation |
| **10** | 6 months* - 4 years** | Cherington, 1974 | USA | M, 52 years | Hospital | Six months after discharge he was able to return to work. After one year, he was examined and believed to be normal. 4 years after symptom onset he continued to work, however, he had some mild residual symptoms (dyspnoea and severe dryness of the mouth). Results of neurological examination were entirely normal as were findings from pulmonary function studies. Repetitive ulnar nerve studies revealed no defect of neuromuscular transmission. | Clinical presentation; Epidemiological data |
| **11** | 4 years** | Cherington, 1974 | USA | F, 48 years | Hospital | Dry mouth, constipation, and fatigue. None of these symptoms has changed in the past two or three years. Neurological examination findings were within normal limits. Repetitive ulnar nerve studies revealed no defect of neuromuscular transmission. | Clinical presentation; Epidemiological data; EMG |
| **12** | 9 years** | Cherington, 1974 | USA | F, 17 years | Hospital | No sequelae | Clinical presentation; Epidemiological data;  Serum sample |
| **13** | 9 years** | Cherington, 1974 | USA | M, 17 years | Hospital | No sequelae | Clinical presentation; Epidemiological data;  Serum sample |
| **14** | 9 years** | Cherington, 1974 | USA | F, 16 years | NR | No sequelae | Clinical presentation; Epidemiological data;  Serum sample |
| **15** | 9 years** | Cherington, 1974 | USA | F, 17 years | NR | No sequelae | Clinical presentation; Epidemiological data;  Serum sample |
| **16** | NR | Oliveira et al., 2022 | Brazil | F, 1,5 years | Hospital | Ataxic gait post-discharge | Clinical presentation; EMG |
| **17** | NR | Paust, 1971 | USA | M, 10 years | ICU | No sequelae | Serum samples; Epidemiological data; Clinical presentation |
| **Infant botulism** | | | | | | | |
| **1** | 1 month* | Schmidt et al., 1992 | USA | F, 1 week | Hospital | Mild hypotonia, poor oral feeding, and gastric tube placement | Stool and serum samples Clinical findings |
| **2** | 1 month | Sabatini et al., 2015 | Italy | M, 4 months | Mixed (hospital, ICU) | No sequelae | Stool sample |
| **3** | 2 months* | Mitchell and Tseng-Ong, 2005 | USA | M, 34 days | ICU | No sequelae | Clinical presentation; Stool sample |
| **4** | 2 months* | Schmidt et al., 1992 | USA | M, 4 months | Hospital | Mild weakness | Stool sample Clinical findings |
| **5** | 2 months* | Mitchell and Tseng-Ong, 2005 | USA | M, 20 days | ICU | Minimal neck extensor weakness, which has resolved at the next visit | Clinical presentation; Stool sample |
| **6** | 5 months* | Schmidt et al., 1992 | USA | M, 9 days | Hospital | Persistent hypotonia and delay in gross motor development | Stool sample Clinical findings |
| **7** | 5 months* | Wohl and Tucker, 1992 | USA | F, 4.5 month | Hospital | No airway sequelae | NR |
| **8** | 1 year* | Mitchell and Tseng-Ong, 2005 | USA | M, 6 weeks | ICU | No sequelae: complete resolution of all signs and symptoms with normal development | Clinical presentation; Stool sample |
| **9** | 1-10 years* | Wohl and Tucker, 1992 | USA | M, 1.5 month | Hospital | No airway sequelae | NR |
| **10** | 1-10 years* | Wohl and Tucker, 1992 | USA | M, 7.5 month | Hospital | No airway sequelae | NR |
| **11** | 1-10 years* | Wohl and Tucker, 1992 | USA | F, 7 months | Hospital | No airway sequelae | NR |
| **12** | 1-10 years* | Wohl and Tucker, 1992 | USA | M, 5.5 months | Hospital | No airway sequelae | NR |
| **13** | 1-10 years* | Wohl and Tucker, 1992 | USA | F, 3 months | Hospital | No airway sequelae | NR |
| **14** | 1-10 years* | Wohl and Tucker, 1992 | USA | M, 4.5 months | Hospital | No airway sequelae | NR |
| **15** | 1-10 years* | Wohl and Tucker, 1992 | USA | F, 5 months | Hospital | No airway sequelae | NR |
| **16** | 1-10 years* | Wohl and Tucker, 1992 | USA | F, 1.2 months | Hospital | No airway sequelae | NR |
| **17** | 1-10 years* | Wohl and Tucker, 1992 | USA | F, 1.5 months | Hospital | No airway sequelae | NR |
| **18** | 1-10 years* | Wohl and Tucker, 1992 | USA | F, 2 months | Hospital | No airway sequelae | NR |
| **19** | NR | Clemmens et al., 2007 | USA | M, 2 months | ICU | No sequelae: fairly rapid recovery | Stool sample |
| **20** | NR | Abdulla et al., 2012 | UK | F, 3 months | Hospital | No sequelae: completely recovered; follow-up assessment showed baby growing and developing normally | Stool sample |
| **21** | NR | Clemmens et al., 2007 | USA | F, 4 months | Hospitalised | No sequelae: gradual but full recovery | Stool sample |
| **22** | NR | Clemmens et al., 2007 | USA | F, 4 months | Hospital | No sequelae: a full recovery over several months | Stool sample |
| **23** | NR | Clemmens et al., 2007 | USA | M, 6 months | Hospital, ICU | No sequelae: a slow but complete recovery | Stool sample, Electromyography |
| **24** | NR | Hurst et al., 1993 | USA | F, 11 days | Hospital | No sequelae: normal tone and was feeding well, gaining weight, and developing normally | Clinical presentation; EMG; Stool sample |
| **Wound botulism** | | | | | | | |
| **1** | 2 months** | Oliveira et al., 2022 | Brazil | F, 4 years | ICU | No sequelae | Clinical presentation; EMG |
| **Adult intestinal toxaemia** | | | | | | | |
| **1** | 3 weeks* - 3.5 months** | Gendrot et al., 2020 | France | F, 26 years | Hospital | Fatigue, orthostatic hypotension, reflex tachycardia. Hypotension was partially improving with the reappearance of the reflex tachycardia and a prolonged sitting position possible. At 3.5 an examination showed an improvement in fatigue, absence of orthostatic hypotension, and complete resolution of neurological sequelae | Serum sample, stool sample |

F – female, M – male, * – follow up period since discharge, ** – follow up period since symptom onset, NR ­­– not reported.

Table S3. Critical Appraisal Skills Programme (CASP) Checklists for Case-Control Studies results.

| **CASE-CONTROL** | **Gottlieb et al., 2007** | **Wilcox et al., 1989** |
| --- | --- | --- |
| 1. Did the study address a clearly focused issue? | Yes | Yes |
| 2. Did the authors use an appropriate method to answer their question? | Yes | Yes |
| 3. Were the cases recruited in an acceptable way? | Yes | No |
| 4. Were the controls selected in an acceptable way? | Yes | Yes |
| 5. Was the exposure accurately measured to minimise bias? | No | Can't Tell |
| 6. (a) Aside from the experimental intervention, were the groups treated equally? | This is an observational study. Only cases have been treated while controls were healthy individuals. | This is an observational study. Only cases have been treated while controls were healthy individuals. |
| 6. (b) Have the authors taken account of the potential confounding factors in the design and/or in their analysis? | Yes | No |
| 7. How large was the treatment effect? | Treatment outcomes were not assessed in this study. | Treatment outcomes were not assessed in this study. |
| 8. How precise was the estimate of the treatment effect? | Treatment outcomes were not assessed in this study. | Treatment outcomes were not assessed in this study. |
| 9. Do you believe the results? | Yes | No |
| 10. Can the results be applied to the local population? | Can't Tell | No |
| 11. Do the results of this study fit with other available evidence? | Yes | Yes |

Table S4. Critical Appraisal Skills Programme (CASP) Checklists for Cohort Studies results.

| **COHORT** | **Tseng-Ong et al., 2007** | **Townes et al., 1996** | **Cohen et al., 1988** | **Schmidt-Nowara et al., 1983** | **Mann et al., 1981** | **Vanella De Cuetos et al., 2011** | **Boccagni et al., 2021** |
| --- | --- | --- | --- | --- | --- | --- | --- |
| 1. Did the study address a clearly focused issue? | Yes | Yes | Yes | Yes | Yes | Yes | Yes |
| 2. Was the cohort recruited in an acceptable way? | Yes | Yes | Yes | No | No | No | No |
| 3. Was the exposure accurately measured to minimise bias? | Yes | Yes | Can't Tell | Yes | Yes | Yes | Yes |
| 4. Was the outcome accurately measured to minimise bias? | Can't Tell | Yes | Yes | Yes | No | No | No |
| 5. (a) Have the authors identified all important confounding factors? | No | No | No | No | No | No | No |
| 5. (b) Have they taken account of the confounding factors in the design and/or analysis? | No | No | Yes | No | No | No | No |
| 6. (a) Was the follow up of subjects complete enough? | Can't Tell | Can't Tell | Yes | Yes | Yes | Can't Tell | No |
| 6. (b) Was the follow up of subjects long enough? | Can't Tell | Yes | Yes | Yes | Yes | No | No |
| 7. What are the results of this study? | Results of the study are described in Table 1. | Results of the study are described in Table 1. | Results of the study are described in Table 1. | Results of the study are described in Table 1. | Results of the study are described in Table 1. | Results of the study are described in Table 1. | Results of the study are described in Table 1. |
| 8. How precise are the results? | Observational studies do not provide any causal relationship. | Observational studies do not provide any causal relationship. | Observational studies do not provide any causal relationship. | Observational studies do not provide any causal relationship. | Observational studies do not provide any causal relationship. | Observational studies do not provide any causal relationship. | Observational studies do not provide any causal relationship. |
| 9. Do you believe the results? | Can't Tell | Yes | Yes | Yes | Yes | Can't Tell | Can't Tell |
| 10. Can the results be applied to the local population? | Can't Tell | No | Yes | Can't Tell | Can't Tell | Can't Tell | Can't Tell |
| 11. Do the results of this study fit with other available evidence? | Yes | Yes | Yes | Yes | Yes | Yes | Yes |
| 12. What are the implications of this study for practice? | Can't Tell | Can't Tell | Can't Tell | Can't Tell | Can't Tell | Can't Tell | Can't Tell |

Table S5. Most frequently reported foodborne botulism sequelae across the studies.

| Outcomes | Boccagni et al., 2021 | | Schmidt-Nowara et al., 1983 | | Mann et al., 1981 | | | | | | Wilcox et al., 1989 | | Gottlieb et al., 2007 | |
| --- | --- | --- | --- | --- | --- | --- | --- | --- | --- | --- | --- | --- | --- | --- |
|  | 73-80 days | | 1 year | | 9 months | | 13 months | | 2 years | | 2 years | | 4.3 years  (range 0.6–6.4 years) | |
|  | S | T | S | T | S | T | S | T | S | T | S | T | S | T |
| Dry mouth | 0 | 4 | 18 | 28 | 12 | 27 | 7 | 14 | 7 | 21 | 5 | 13 | 32 | 211 |
| Dyspnea | 0 | 4 | 13 | 28 | 13 | 27 | 7 | 14 | 9 | 21 | 12 | 13 | 38 | 211 |
| Weakness | 2 | 4 | NR | NR | 12 | 27 | 8 | 14 | 12 | 21 | 10 | 13 | 91 | 211 |
| Constipation | NR | NR | 10 | 28 | 6 | 27 | 4 | 14 | 3 | 21 | 4 | 13 | NR | NR |
| Difficulty swallowing | 1 | 4 | NR | NR | 3 | 27 | 2 | 14 | 3 | 21 | 2 | 13 | NR | NR |
| Limitations in vigorous activities/ Exercise intolerance | NR | NR | NR | NR | 15 | 27 | 9 | 14 | 10 | 21 | NR | NR | 135 | 211 |
| Blurred vision | NR | NR | NR | NR | 5 | 27 | 3 | 14 | 3 | 21 | 6 | 13 | NR | NR |
| Diplopia | 1 | 4 | NR | NR | 3 | 27 | 1 | 14 | 3 | 21 | 0 | 13 | NR | NR |
| Fatigue | NR | NR | 19 | 28 | NR | NR | NR | NR | NR | NR | 11 | 13 | 101 | 211 |
| Dizziness | NR | NR | NR | NR | NR | NR | NR | NR | NR | NR | 5 | 13 | 55 | 211 |

S – individuals with persistent symptoms, T – total number of participants, NR – not reported.
